# Disease-modifying drug initiation patterns in commercially insured multiple sclerosis patients: a retrospective cohort study

**DOI:** 10.1186/1471-2377-11-122

**Published:** 2011-10-06

**Authors:** Jay M Margolis, Robert Fowler, Barbara H Johnson, Cheryl A Kassed, Kristijan Kahler

**Affiliations:** 1 Thomson Reuters, 332 Bryn Mawr Ave., Bala Cynwyd, PA, 19004, USA; 2 Thomson Reuters, 4301 Connecticut Ave NW, Suite 330, Washington, DC, 20008, USA; 3 Health Analytics, LLC, 9200 Rumsey Road, Suite 215, Columbia, MD, 21045, USA; 4 Novartis Pharmaceuticals, One Health Plaza, USEH 501/399O, East Hanover, NJ, 07936, USA

## Abstract

**Background:**

The goal of this research was to compare the demographics, clinical characteristics and treatment patterns for newly diagnosed multiple sclerosis (MS) patients in a commercial managed care population who received disease-modifying drug (DMD) therapy versus those not receiving DMD therapy.

**Methods:**

A retrospective cohort study using US administrative healthcare claims identified individuals newly diagnosed with MS (no prior MS diagnosis 12 months prior using ICD-9-CM 340) and ≥ 18 years old during 2001-2007 to characterize them based on demographics, clinical characteristics, and pharmacologic therapy for one year prior to and a minimum of one year post-index. The index date was the first MS diagnosis occurring in the study period. Follow-up of subjects was done by ICD-9-CM code identification and not by actual chart review. Multivariate analyses were conducted to adjust for confounding variables.

**Results:**

Patients were followed for an average of 35.7 ± 17.5 months after their index diagnosis. Forty-three percent (n = 4,462) of incident patients received treatment with at least one of the DMDs during the post-index period. Treated patients were primarily in the younger age categories of 18-44 years of age, with DMD therapy initiated an average of 5.3 ± 9.1 months after the index diagnosis. Once treatment was initiated, 27.7% discontinued DMD therapy after an average of 17.6 ± 14.6 months, and 16.5% had treatment gaps in excess of 60 days.

**Conclusions:**

Nearly 60% of newly-diagnosed MS patients in this commercial managed care population remained untreated while over a quarter of treated patients stopped therapy and one-sixth experienced treatment gaps despite the risk of disease progression or a return of pre-treatment disease activity.

## Background

Multiple sclerosis (MS) is a complex, recurrent, and progressive autoimmune disease of the central nervous system affecting an estimated 250,000 to 400,000 people in the US. Primarily diagnosed in young adults at a mean age of 29 years, it follows four recognized disease courses: relapsing/remitting MS (RRMS), secondary progressive MS (SPMS), primary progressive MS (PPMS), and progressive relapsing MS (PRMS) [1-4]. Approximately 20% of patients have a mild form of the disease, yet MS can also render a person unable to write, speak, or walk, with up to 60% of patients losing ambulatory capability within 20 years after onset [1,2,5]. Since most people with MS have a fairly normal life expectancy, this chronic disease evokes very significant social, medical and economic impacts.

MS is neither easy nor quick to diagnose, especially in its early stages, compounded by the often transient nature of attacks, variability in symptoms, other diseases producing similar symptoms, and the lack of a specific unequivocal diagnostic test [1-4,6,7]. Clinical diagnosis often requires several strategies to determine if a person meets the established criteria (e.g., The Revised McDonald Criteria), including a detailed medical history, neurologic exams, magnetic resonance imaging (MRI), evoked potentials (EP) and spinal fluid analysis [7-9]. Symptoms may vary in frequency, severity and form, and so patients diagnosed with clinically definite MS will typically have been through several diagnostic stages often drawn out over months or years [10-17].

There is presently no known cure for MS. Current treatments for MS are aimed at returning function after an attack, preventing new attacks, managing symptoms and preventing or postponing long-term disability [18]. Acute symptomatic attacks are most often treated with high dose glucocorticoids, however there does not appear to be any long-term functional benefit from their use [18,19]. Several disease-modifying drugs (DMDs) have been found to reduce the relapse rate, reduce lesion development, positively affect MRI metrics and disease progression, and improve the quality of life for many MS patients [3,18,20-23]. Six parenterally administered DMDs are currently approved for immunomodulatory and immunosuppressive treatment of relapsing and secondary-progressive MS (in alphabetical order): glatiramer acetate, interferon beta 1a--intramuscular, interferon beta 1a--subcutaneous, interferon beta 1b, mitoxantrone, and natalizumab [21]. DMD treatment can be sustained indefinitely, as long as there is evidence of benefit, particularly since cessation of therapy may result in returning to pre-treatment disease activity [21,24-29].

The decision to begin or continue MS therapy is complex and controversial [5]. The National Multiple Sclerosis Disease Management Consensus Statement [21] recommends initiating interferon beta or glatiramer DMD therapy as soon as possible following definite diagnosis of active, relapsing MS, and also to consider DMD therapy for selected high risk patients with a first attack. Although the DMDs have positively affected the treatment and course of RRMS, numerous factors including their parenteral route of administration, injection anxiety, side effects, unobservable improvement, adjunctive therapy, and treatment fatigue can affect the initiation and continuation of DMD therapies [5,17,23,25-29].

The objective of the current research was to understand the differences in demographics, clinical characteristics and treatment patterns between newly diagnosed MS patients in a commercial managed care population who received DMD therapy versus those not receiving DMD therapy.

## Methods

### Study design

This retrospective cohort study used administrative healthcare claims from a database of US-based employers and health insurers to identify individuals newly diagnosed with MS during 2001-2007 and to construct a detailed claims history for each individual. Patients were characterized based on their demographics and baseline clinical indicators (diagnoses, comorbidities, concomitant medications) during the year prior to the index diagnosis, and then followed for at least one year to assess healthcare resource utilization, medical expenditures, treatments, and adherence to therapy.

### Data source

Data came from two Thomson Reuters MarketScan^® ^research databases: the Commercial Claims and Encounters (Commercial) database and the Medicare Supplemental and Coordination of Benefits (Medicare) database, from January 1, 2000, through December 31, 2007. The Commercial database contained the inpatient, outpatient, and outpatient prescription drug experience of 44.5 million employees and their dependents under a variety of insurance coverage types. The Medicare database contained the healthcare experience of 4.5 million individuals with Medicare supplemental insurance paid for by employers, including both the Medicare-covered portion of payment and the employer-paid portion. All database records were de-identified and fully compliant with US patient confidentiality requirements (HIPAA).

### Sample selection and study period

Selected patients were a minimum of 18 years old with a diagnosis of MS (ICD-9-CM code 340) between January 1, 2001, and January 1, 2007 (patient selection period). A minimum of one inpatient or emergency department claim, or more than one outpatient claim at least 30 days apart with ICD-9-CM 340 (excluding diagnostic laboratory and diagnostic radiology claims; also referred to herein as "non-diagnostic" claims) was required. The index diagnosis date was assigned to the earlier of the first medical claim with an MS diagnosis or the date of the first medical or pharmacy claim for one of the DMDs during the patient selection period. Patients were required to have at least 12 months of continuous health plan eligibility with both medical and pharmacy benefits both before and following their index diagnosis date. To identify patients as "incident" or newly diagnosed, they were required to have no claims with a diagnosis of MS or any DMD therapy during the 12-months prior to their index diagnosis date. The study used administrative claims data in the absence of the patient medical charts, therefore relying on the diagnoses coded by the provider and the patient history found only within the healthcare claims. Patients were followed for a minimum of 12 months from their index date and then for as long as the data were available or until the end of calendar year 2007. Follow-up of subjects was done by ICD-9-CM code identification and not by actual chart review.

### Outcome measures

Diagnoses, medical procedures and pharmacologic therapy were derived from medical and pharmacy claims received according to service dates in the patient's pre-index and post-index periods. MS diagnoses and comorbidities were determined from medical claims (excluding diagnostic laboratory and radiology claims). A diagnosis code entered in any position for the claim was used. Pharmacologic therapy was identified from pharmacy claims and from HCPCS codes in medical claims. Medical procedures or diagnostic evaluations were identified from CPT-4 codes or ICD-9-CM procedure codes in medical claims. Drugs for treating MS were analyzed individually and by drug class.

Demographic data included age, gender, insurance plan type (comprehensive, EPO, HMO, POS, POS with capitation, PPO, and other), geographic region and length of follow-up. Clinical data included monitoring for selected comorbidities in medical claims, computation of the Deyo-Charlson comorbidity index (measured pre-index), use of DMD therapy, other pharmacological treatments for MS symptoms, and medical procedures and diagnostic tests associated with MS.

Treatment pattern analyses included time to DMD therapy initiation (for patients treated), persistence on therapy and discontinuation patterns. The length of continuous treatment with the index DMD was determined by considering the days supplied listed on the index drug claim(s), as well as the fill date and days supplied for subsequent claims of the same medication, including medical claims showing DMD administration. The length of index DMD therapy was defined as "continuous" by calculating days across consecutive prescription or medical claims of the index drug with no gaps of more than 30 days. A gap between consecutive prescriptions of the index drug is typically allowed because patients may not fill prescriptions the exact day they are due for a refill. The number of patients with gaps in therapy of 31-60 days, 61-90 days, 91-120 days and >120 days were also tracked. Gaps for patients receiving natalizumab and mitoxantrone were adjusted to accommodate for their longer dosing intervals, assuming 4 weeks for each medical claim date for natalizumab and 12 weeks from each medical claim date for mitoxantrone [30,31]. As a part of the gap analysis, the number of patients with evidence of the index DMD after a gap of >60 days ("restarts") were identified and the average number of days to restart was measured. The length of DMD therapy from the first until the last drug claim of the index DMD was also measured. This measure counted the number of days between the treatment index date (i.e., the first drug claim for the index medication) and the last drug claim for the index medication (including days of supply) during the study period, regardless of gaps in therapy. The length of continuous treatment with all DMD therapy was measured, both with and without gaps of greater than 30 days. For each patient's drug regimen, the time from the first DMD claim to the date of the last applicable DMD claim (including days of supply) was measured, including any DMD therapy that was received. Switching among DMDs and discontinuation of all DMDs (i.e., no evidence of any DMD for a 60-day period following the end of the last observed prescription) was also analyzed.

### Statistical analysis

Categorical variables were presented as counts and proportions. Continuous variables were summarized by providing the number of observations, the mean, the standard deviation, the median, and the range. Statistical tests of significance for differences in these distributions included Chi-square tests to evaluate the statistical significance of differences for categorical variables, t-tests and ANOVA for normally distributed continuous variables, while nonparametric Wilcoxon and Kruskal-Wallis tests were used for continuous variables that are not normally distributed. P-values of 0.050 or less were considered statistically significant.

Multivariate regressions were conducted to control for imbalances in observed baseline demographics and clinical characteristics. Cox proportional hazards regressions were used to assess characteristics associated with treatment initiation with adjustment for time from diagnosis to first treatment as well as to assess treatment persistence, measured as the time to a 31 day gap in therapy, among those treated. A logistic regression model was also used to predict patient characteristics affecting the initiation of treatment with DMDs. For each of the three models, age and gender were included as covariates and a backward elimination method was employed to select among the many other characteristics, with the variables remaining in each model each statistically significant at a level of 5%. All statistical analyses were performed using SAS version 9.2.

## Results

### Sample Attrition

Initially there were 67,869 Commercial and Medicare patients with at least one inpatient or two non-diagnostic outpatient claims at least 30 days apart with an MS diagnosis between January 1, 2001 and January 1, 2007. Of these, 34,699 (51.1%) were excluded after applying twelve months pre- and post-index continuous eligibility requirements, 306 (0.5%) were excluded who were under the age of 18, and 21,803 patients (32.1%) were excluded who had an MS diagnosis or DMD treatment during the pre-index period, resulting in a final cohort of 11,061 patients.

### Demographics

Population demographics are shown in Table 1. Females comprised 74.2% of the study population. The average length of follow up was 35.7 ± 17.5 months. There were 40.3% (n = 4,462) of patients treated with at least one of the DMDs during the study period. The mean population age was 50.3 ± 13.3 years, with DMD-treated patients significantly younger (mean age 44.4 ± 10.6 years) than those without DMD treatment (mean age 54.3 ± 13.4 years, p < 0.001). The age distributions show skewing of treatment to younger age groups, with 48.8% of DMD-treated patients under the age of 44 compared with 23.3% of untreated patients, and 2.2% of patients over 65 with DMD treatment versus 20.7% of untreated patients.

**Table 1:** Patient Population Demographics

Demographic	All patients	DMD-treated	Not DMD treated
	N = 11,061	n = 4,462	n = 6,599

Gender			

Male	25.8%	23.7%	27.2%

Female	74.2%	76.3%	72.8%

Age (in years, mean ± SD)	50.3 ± 13.3	44.5 ± 10.6	54.3 ± 13.4

18-34	11.5%	18.4%	6.8%

35-44	21.5%	30.4%	15.5%

45-54	31.2%	33.5%	29.7%

55-64	22.5%	15.6%	27.2%

65+	13.2%	2.2%	20.7%

Insurance Type			

Commercial	85.1%	96.9%	77.1%

Medicare	14.9%	3.1%	22.9%

Geographic Region			

Northeast	12.9%	12.8%	13.0%

North Central	35.6%	32.0%	38.0%

South	30.4%	35.5%	27.0%

West	20.5%	19.2%	21.3%

Unknown	0.6%	0.5%	0.7%

Index year			

2001	7.5%	8.0%	7.2%

2002	12.9%	13.0%	12.9%

2003	16.8%	17.7%	16.1%

2004	24.0%	22.1%	25.4%

2005	22.2%	22.5%	21.9%

2006*	16.6%	16.7%	16.5%

Length of follow-up (in months, mean ± SD)	35.7 ± 17.5	36.7 ± 17.6	35.1 ± 17.5

*Patients who indexed on January 1, 2007 are included in 2006 category

### Clinical characteristics

Chronic pain conditions were the most frequently noted comorbidities in the medical claims of this MS population (Table 2), with over half of the cohort reporting chronic skeletal pain (joint derangements, dorsopathies, or polymyalgia rheumatica). With the exception of fatigue, headache, depression, skin cancers, and sleep disorders, all other comorbidities were noted in significantly higher percentages of patients not treated with DMDs. The Deyo-Charlson Comorbidity Index was calculated for all patients, measured using pre-index comorbidities, and was significantly lower for DMD-treated patients, computed at 0.36 ± 0.86 for DMD-treated patients and 0.64 ± 1.24 for untreated patients, p < 0.001.

**Table 2:** MS Comorbidities and Pharmacologic Treatments of Interest During the Post-Index Period

Comorbidity*	DMD-treated (n = 4,462)	Not DMD treated (n = 6,599)	p-value
Joint derangement, dorsopathies, polymyalgia rheumatica	52.7%	57.1%	<0.001

High blood pressure	30.5%	40.7%	<0.001

High cholesterol	23.2%	28.6%	<0.001

Cancers excluding skin cancer	22.2%	28.2%	<0.001

Fatigue	25.1%	24.1%	0.231

Urinary tract infections	21.6%	25.9%	<0.001

Headache	22.0%	21.9%	0.897

Neuropathic pain	19.6%	22.7%	<0.001

Depression	21.0%	18.6%	0.002

Anxiety	18.3%	19.8%	0.049

Arthritis (RA & OA)	12.6%	21.4%	<0.001

Skin cancers	13.8%	14.9%	0.107

Thyroid disease	12.2%	15.1%	<0.001

Diabetes	8.8%	14.8%	<0.001

Sleep disorders	11.5%	11.0%	0.469

Index DMD**			

Glatiramer acetate	33.7%		

Interferon beta-1a IM	32.1%		

Interferon beta-1a SQ	20.5%		

Interferon beta-1b	12.7%		

Mitoxantrone	0.6%		

Natalizumab	0.4%		

Other medications used for treating MS exacerbations			

Corticosteroids	61.4%	40.0%	<0.001

Methotrexate	1.4%	2.0%	0.020

Azathioprine	1.3%	1.0%	0.165

Cyclosporine	1.2%	1.4%	0.336

Mycophenolate	1.1%	0.7%	0.027

IVIG	1.3%	0.6%	<0.001

Cyclophosphamide	0.6%	0.5%	0.416

Muscle relaxants	37.6%	33.6%	<0.001

Antidepressants	37.9%	30.4%	<0.001

Anti-fatigue agents	32.7%	12.5%	<0.001

*ICD-9-CM codes used for these conditions are available on request.

**First DMD on or after the patient's index date.

### Treatment patterns

The most common index DMD used in treating these incident patients was glatiramer (33.7% of treated incident patients) followed by interferon-beta-1a IM (32.1%) and interferon-beta-1a SQ (20.5%) (Table 2). The start of availability for interferon beta-1a SQ in the US in February, 2002 is acknowledged as a factor possibly underestimating its relative reported usage over the five-year time span of this study. Similarly, it is noted that natalizumab was approved by the US FDA in November 2004, withdrawn in February 2005, and then re-approved for relapsing MS in June 2006.

Other drugs used for acute MS exacerbations but without established evidence of altering the course of disease progression are also shown in Table 2. Glucocorticoids usage was highest in patients receiving DMDs post-index (61.4%) compared with patients who were not treated with DMDs post-index (40.0%). Usage of the other agents was generally in 2% or fewer of patients. No patients were found in this timeframe who had received cladribine, daclizumab, or anti-lymphocyte globulin post-index.

Muscle relaxants, antidepressants, and anti-fatigue agents were frequently prescribed post-index in this population, also shown in Table 2, particularly among DMD-treated patients whose percentage of patients with drug use was significantly higher for all three of these drug classes compared to untreated patients (p < 0.001). Pre-index use of these medications was significantly more prevalent in patients who did not receive DMD treatment (muscle relaxants 16.1% DMD vs. 20.8% non-DMD, p < 0.001; antidepressants 20.3% DMD vs. 22.2 non-DMD, p = 0.018; anti-fatigue agents 3.5% DMD vs. 5.8% non-DMD, p < 0.001).

The mean time to therapy initiation was 5.3 ± 9.1 months following the index diagnosis (Table 3). Once started on their index medication, more than three-quarters (78.7%) of these patients stayed on their index medication without switching to another DMD for the duration of the follow-up period. Nearly three-quarters of patients (71.0%) stayed on their index therapy an average of 17.0 ± 15.9 months without gaps exceeding 30 days. Including the 21.3% of patients who switched to another DMD after an average of 14.8 ± 12.9 months on their index DMD, patients appeared to remain on all DMD therapy without gaps exceeding 30 days for an average of 18.8 ± 16.8 months. (Table 3)

**Table 3:** Pharmacotherapy Treatment Persistence Patterns

Persistence Measure	DMD-treated patients (n = 4,462)
Persistence on DMD therapy	Mean ± SD

Days from index diagnosis to 1^st ^DMD	158 ± 274

Days of continuous therapy for index DMDs (no gaps > 30 days)	511 ± 476

Days of continuous therapy for all DMDs (no gaps > 30 days)	565 ± 503

Days, first to last for index DMD (with gaps)*	683 ± 525

Days, first to last for all DMDs (with gaps)*	798 ± 540

Gap analysis for the index DMD	% of patients

No gaps in index therapy > 30 days	71.0%

Gaps in index therapy of 31-60 days	20.6%

Gaps in index therapy > 60 days (restarts)	16.5%

Gaps of 61-90 days	8.4%

Gaps of 91-120 days	4.4%

Gaps > 120 days	8.3%

Mean days to restart (Mean ± SD)	159 ± 151

Switched from index DMD	21.3%

Mean days to switch (Mean ± SD)	445 ± 386

Discontinued all DMDs before end of follow-up†	27.7%

Mean days to discontinuation (Mean ± SD)	527 ± 437

There were 29.0% of patients with gaps in therapy exceeding 30 days, and over 16% with gaps in excess of 60 days (restarting therapy), as shown in Table 3. Overall, 27.7% of patients discontinued DMD therapy altogether after an average of 17.6 ± 14.6 months without restarting any DMD therapy.

Factors with statistically significant associations with the initiation of DMD therapy are shown in Table 4. The hazard ratio denotes the odds that one group of patients was treated with a DMD compared with a reference group of patients, controlling for the influence of other variables in the model and the length of follow-up. Those variables associated with an increased likelihood of initiating DMD therapy include patient age (the lower the patient age, the higher the odds of being treated), evidence of loss of coordination, and the occurrence of an NMRI or spinal tap prior to the index diagnosis. Factors associated with a decreased likelihood of DMD initiation include higher degrees of comorbidity (per the Deyo-Charlson Comorbidity Index showing a 13 percent decrease for each point rise in the index), headache, sleep disorders, or the occurrence of pressure ulcers prior to the index diagnosis, as well as the pre-index use of azathioprine, anti-fatigue medications, or muscle relaxants.

**Table 4:** Factors Associated with Initiation of DMD Therapy

Parameter	Hazard Ratio	Pr > ChiSq	Standard Error
Gender (reference: male)			

Female	0.998	0.949	0.037

Age group (reference: 18-34)			

Age 35-44	0.845	<0.001	0.045

Age 45-54	0.641	<0.001	0.045

Age 55-64	0.424	<0.001	0.055

Age 65+	0.107	<0.001	0.112

Pre-index comorbidities			

Deyo-Charlson Comorbidity Index	0.867	<0.001	0.020

Loss of coordination	1.537	<0.001	0.080

Headache	0.797	<0.001	0.042

Sleep disorders	0.781	0.002	0.079

Pressure ulcer	0.225	0.003	0.501

Pre-index pharmacotherapy			

Anti-fatigue agent use	0.824	0.018	0.082

Azathioprine use	0.289	0.003	0.410

Corticosteroid use	1.081	0.031	0.036

Muscle relaxant use	0.823	<0.001	0.042

Pre-index testing			

Electrocardiogram	0.883	0.001	0.038

Nuclear MRI	2.361	<0.001	0.035

Spinal tap	1.819	<0.001	0.043

* Table was created using a Cox Model adjusting for time to DMD initiation. The dependent variable was days to DMD initiation. Shown in this table are those factors of statistical significance (except gender). There were more parameters modeled in each category and others eliminated using backward selection.

Factors with statistically significant associations with the persistence on DMD therapy are shown in Table 5. Those associated with an increased likelihood of persisting on DMD therapy include female gender, age of 18 to 34, having an HMO insurance plan type, higher degrees of comorbidity (Deyo-Charlson Comorbidity Index), pre-index comorbidities of depression, headache, or urinary tract infection, and pre-index use of antidepressants or muscle relaxants. Factors associated with a decreased likelihood of DMD persistence include age greater than 34 and pre-index neuropathic pain.

**Table 5:** Factors Associated with Persistence on DMD Therapy

Parameter	Hazard Ratio	Pr > ChiSq	Standard Error
Gender (reference: Male)			

Female	1.117	0.035	0.052

Age group (reference: Age 18-34)			

Age 35-44	0.701	<0.001	0.060

Age 45-54	0.618	<0.001	0.061

Age 55-64	0.672	<0.001	0.073

Age 65+	0.847	0.274	0.152

Insurance plan type (reference: Comprehensive)			

HMO	1.305	0.001	0.079

PPO	0.972	0.739	0.084

POS	1.096	0.170	0.067

POS with Capitation	1.002	0.984	0.109

Pre-index comorbidities			

Deyo-Charlson Comorbidity Index	1.049	0.045	0.024

Depression	1.273	0.001	0.074

Headache	1.162	0.008	0.056

Neuropathic pain	0.844	0.003	0.058

Urinary tract infection	1.206	0.010	0.072

Pre-index pharmacotherapy			

Antidepressant use	1.137	0.018	0.055

Muscle relaxant use	1.175	0.005	0.057

* This table was created using a Cox Model adjusting for time to DMD Therapy Gap of 31+ Days. The dependent variable was days to DMD therapy gap of 31+ days. Shown in this table are those factors of statistical significance. There were more parameters modeled in each category and others eliminated using backward selection.

## Discussion

This study investigated the demographics, clinical characteristics, and treatment patterns for DMD therapy in an adult managed care population of newly diagnosed MS patients. Patients were followed for an average of 35.7 ± 17.5 months after their index diagnosis. The results indicate that 40.3% of incident patients received treatment with at least one of the DMDs during the post-index period. Population demographics were similar to a prior study done in a managed care population [32]. Treated patients were primarily in the younger age categories of 18-44 years of age, with DMD therapy initiated an average of 5.3 ± 9.1 months after the index diagnosis. Patients most likely to receive DMD therapy were under the age of 45, had evidence of loss of coordination, and had received NMRI or spinal tap pre-index. Once treatment was initiated, 72.3% of DMD-treated patients remained on therapy for the remainder of their study period.

In the age categories of 18-34 and 35-44, most patients received DMD treatment (64.7% and 57.0% respectively), while most patients in the upper age categories did not receive DMD treatment (43% in ages 45-54; 27.8% in ages 55-64; and, 6.6% in ages 65+). The reasons for the increasing percentages of untreated patients in the older groups are unknown, but disease severity and disease stage are suspected contributors to the decision not to opt for treatment. The use of retrospective claims data in the absence of the patient's medical chart relies on the provider's coding of an MS diagnosis, and contributes a very limited view of the patient's medical history. It is possible that the window of 12 months without MS claims prior to the first observed MS claim may have resulted in misclassification, particularly for patients with long-standing or advanced disease, those in remission, or other patients no longer seeking treatment. The low percentage of DMD treated patients found in this analysis may be due in part to these limitations and methodological issues. More rigorous confirmation of the accuracy of the diagnosis data were unable to be performed due to patient de-identification requirements of the data sources. Differences in the frequency of comorbidities may also be attributable to the differences in age between the DMD-treated and untreated groups, as higher percentages of patients with arthropathies, hypertension, hypercholesterolemia, cancers, UTIs, thyroid disease and diabetes may be expected in the older (untreated) population. In addition, a diagnosis was required in only one claim for indication of a comorbidity in this study, and so the percentage of patients with comorbidities may be overestimated as some diagnosis coding may have been either rule-outs or miscoding.

Adherence with DMDs can be complicated by many factors [26,33], not the least of which are the concomitant medications that patients may need to take for relief of MS symptoms, such as muscle relaxants or anti-fatigue medications, as well as for comorbidities that occur as a normal part of aging. The comorbidities found post-index in this population are often medicated chronically, such as the arthropathies, hypertension, hyperlipidemia, depression, and neuropathic pain. Multiple chronic medications can impose both a motivational as well as financial burden on the patient.

This study found 71.0% of DMD-treated patients continued their index DMD treatment for 17.0 ± 15.9 months without gaps exceeding 30 days, compared to an adherence rate of 62% to 64% among patients taking insulin for type 2 diabetes and 68% to 79% among patients taking oral medications for heart failure [33]. In this study's cohort of newly diagnosed patients treated with DMDs, there were 27.7% who discontinued DMD therapy after an average of 17.6 ± 14.6 months. Although not directly comparable, Rio et al (2005) found in their cohort of 632 patients followed over 8 years (mean of 47.1 months) that 17% (107) stopped therapy with approximately half of those stopping prior to 2 years [26], and O'Rourke (2005) found a stopping rate for interferon-beta of 28%, as well as an Italian study of RRMS that noted 41% of patients treated with interferon-beta had stopped after three years [28]. Further, this study had 29.0% of patients with gaps in therapy exceeding 30 days, and over 16% with gaps in excess of 60 days. The reasons for discontinuance and non-adherence gaps are not known. Costello et al (2008) explored reasons for non-adherence to MS therapy and found the key barriers to adherence to be problems with injecting, perceived lack of efficacy, adverse events, and treatment fatigue [33]. It has also been reported that patients discontinuing treatment may also represent higher disability or worsening response to the treatment [26-28,33].

There is a growing body of evidence that progression of clinical disease occurs following cessation of DMD therapy [24,25,34]. Drugs that can increase the convenience or tolerability to DMD treatment for MS patients have the potential to increase the rate of early treatment and overall adherence, offering benefit for those who cannot tolerate or adhere to injectable medications [35-44]. The advent of therapies with increased convenience, tolerability and efficacy is indeed encouraging for advancing the treatment of MS.

### Limitations

This study was subject to a number of limitations. Using administrative claims data in the absence of the patient medical charts, and limiting the look-back period to 12 months of pre-index claims history precluded establishing each patient's long term prior disease history. These factors also obscured a definitive MS diagnosis, the type of MS (e.g., relapsing-remitting, primary progressive), identification of the initial date of an MS diagnosis, therapy occurring prior to the start of the study, disease severity, and the occurrence of relapse. Claims analysis is limited in its ability to account for all possible differences in patients and providers residing in different states. In addition, misclassification errors are possible when relying on diagnosis coding from administrative claims data and where the extent of undercoding of diagnoses is unknown. In the absence of diagnosis coding on pharmacy claims, it was assumed that the drugs shown to be used for treating MS were actually used as such. Further, the data sources did not include information on patient assistance programs or investigational drug treatment programs administered outside of a health plan's coverage or benefit plan, and so only treatments submitted to a health plan for coverage were available for analysis. The databases represent primarily employed or retired individuals, and thus may not be generalizable to the entirety of the US MS population.

## Conclusions

The decision to initiate DMD treatment, the choice of therapy, and the extended deliberations to continue treatment are complex. Individualized decisions made by each patient with their provider consider many factors including disease severity, benefits of therapy, comparative effectiveness of available therapies, potential side-effects, and the effects on a patient's budget and lifestyle. This study found that a large percentage of newly-diagnosed MS patients remained untreated during the study period, that at least a quarter of treated patients stopped therapy during the time of this study, and one-sixth of treated patients experienced treatment gaps despite the risk of disease progression or a return to pre-treatment disease activity. New pharmacologic agents with increased convenience, tolerability and efficacy have promise for improving the overall quality of life for MS patients and their families.

## List of Abbreviations

(DMD): disease-modifying drug; (EP): evoked potentials; (EPO): exclusive provider organization; (HMO): health maintenance organization; (MRI): magnetic resonance imaging; (MS): multiple sclerosis; (NMRI): nuclear magnetic resonance imaging; (POS): point of service; (PPMS): primary progressive multiple sclerosis; (PPO): preferred provider organization; (PRMS): progressive relapsing multiple sclerosis; (RRMS): relapsing/remitting multiple sclerosis; (SPMS): secondary progressive multiple sclerosis;

## Competing interests

Jay Margolis, Robert Fowler, Barbara Johnson, and Cheryl Kassed (at the time of the study) are employees of Thomson Reuters (Healthcare & Science), who was paid by Novartis Pharmaceuticals in connection with the development of this manuscript. Kristijan Kahler is an employee of Novartis Pharmaceuticals.

## Authors' contributions

JM participated in the study concept and design, acquisition and analysis of data, interpretation of data, drafting and revision of the manuscript. RF participated in the study design, acquisition and analysis of data, statistical interpretation of the data, and review and revision of the manuscript. BHJ participated in the study design, acquisition and analysis of data, interpretation of data, and review and revision of the manuscript. CK reviewed existing literature, researched and wrote the background section of the manuscript, as well as reviewing and editing of the manuscript. KK conceived of the study and participated in its design, analysis of results, and review and revision of the manuscript. All authors read and approved the final manuscript.

## Pre-publication history

The pre-publication history for this paper can be accessed here:

http://www.biomedcentral.com/1471-2377/11/122/prepub
